# Development of a Novel Rice-Based Snack Enriched with Chicory Root: Physicochemical and Sensory Properties

**DOI:** 10.3390/foods11162393

**Published:** 2022-08-09

**Authors:** Jelena Bokić, Jovana Kojić, Jelena Krulj, Lato Pezo, Vojislav Banjac, Dubravka Škrobot, Vesna Tumbas Šaponjac, Strahinja Vidosavljević, Viktor Stojkov, Nebojša Ilić, Marija Bodroža-Solarov

**Affiliations:** 1 Faculty of Technology, University of Novi Sad, Bul. Cara Lazara 1, 21000 Novi Sad, Serbia; 2 Institute of Food Technology, University of Novi Sad, Bul. Cara Lazara 1, 21000 Novi Sad, Serbia; 3 Institute of General and Physical Chemistry, University of Belgrade, Studentski Trg 12–16, 11000 Beograd, Serbia

**Keywords:** chicory root, extrusion, characterization, artificial neural network, genetic algorithm, optimization

## Abstract

A novel rice-based snack enriched with chicory root flour (CRF) was developed by twin-screw extrusion. Chicory (*Cichorium intybus* L.) is one of the promising medicinal plants for the development of innovative food and may be considered a functional food ingredient. Central composite design (CCD) was employed to generate snack formulations by varying feed moisture (M, 16.3–22.5%), screw speed (SS, 500–900 rpm) and CRF content (20–40%). The optimization according to artificial neural network modeling and a genetic algorithm was applied to define optimal process conditions (17.6% moisture, 820 rpm and 24.1% of CRF) for obtaining the product with the highest expansion (3.34), crispiness (3.22 × 10^−3^), volume (2040 m^3^), degree of gelatinization (69.70%) and good color properties. Bulk density (110.33 g/L), density (250 kg/m^3^), and hardness (98.74 N) resulted in low values for the optimal sample. The descriptive sensory analysis evaluated low hardness and bitterness, with high crispiness for the optimal extrudate. This study points to the possibility of a novel chicory enriched extrudate production with desirable physicochemical and sensory properties.

## 1. Introduction

Extrusion cooking is a promising food-processing technique able to produce versatile snacks and achieve various shapes and textures of final products [1]. However, commercially available extrudates are generally low in nutritional value, which leads to efforts from the food sector to enrich such products with nutritionally valuable raw materials [2]. In the creation of these new products, innovative food production approaches are aimed to implement food industry by-products, such as fruit and vegetable pomace and bagasse, oilseed cakes, brewers spent grains, and cereal brans and whey to achieve sustainable food production without food waste residues and to improve products quality [3].

Extruded food products are mainly based on cereals rich in starch, such as wheat, corn or rice. Rice (*Oryza sativa* L.) is used as a common food in the human diet or as a raw material for developing value-added products such as snacks, breakfast cereals, modified starches and similar products [4]. Its versatile uses are due to its flavor-carrying capability, hypoallergenicity, mild taste and good processing properties [4].

Many plant materials were incorporated into extrudates, such as yam root flour (*Dioscorea* sp.) [5] and the flours of Jerusalem artichoke, pumpkin, and amaranth [6], to produce innovative snacks with nutritional added value. One of the promising materials for food fortification is chicory (*Cichorium intybus* L.) root flour, rich in polyphenols, minerals, inulin, oligofructose and sesquiterpene lactones considered as potential carriers of food functionality [7]. Pouille et al. [8] reported potential cancer prevention, antibacterial and antiviral defense, and the prebiotic, hypoglycemic and hypolipidemic effects of chicory root flour and its water extract. However, no studies were conducted with the goal of investigating the impact of a chicory root flour (CRF) addition to extrudates and their physicochemical and sensory properties. The application of chicory root in the food industry refers to the roasted and ground root as a substitute or addition to coffee, dried root in teas and tea mixtures, fresh chicory root as a salad, or as a commercial source of inulin [9,10]. Some scientific studies have studied chicory root in the production of crackers [11], yogurt and ice cream [12,13]. However, no studies were conducted to investigate the impact of chicory root flour (CRF) addition on physicochemical and sensory properties of rice-based extrudates.

Developing an innovative food product is especially challenging from the sensory aspect. Extrusion cooking affects consumer acceptance mainly due to snack appearance and textural properties. Relating the extrusion process parameters to the physical and sensory properties of snacks to ensure sensory acceptability is imperative from the technological point of view. The evaluation of the sensory quality of the product can guide the direction of changes in production to make the product appealing to consumers. Optimal process conditions are required for obtaining such a product that satisfies consumer demands, product quality and safety. Mathematical modeling is a powerful tool for adjusting the product′s properties and may predict the characteristics of the final products. Applying a mathematical model can define the production conditions that will give the preferable product properties and thus represent a time-saving and cost-effective method. Artificial neural networks (ANNs) and genetic algorithms (GAs) are widely applied in food extrusion and may be utilized to optimize or predict product [14] or process conditions [15].

This study is aimed to design a novel rice-based snack fortified with CRF to facilitate the implementation of chicory as a promising food ingredient for snack foods. Furthermore, the physicochemical and sensory characterization of developed snacks was conducted in order to define their quality. Moreover, the impacts of extrusion process parameters (feed moisture, screw speed and CRF content) on the properties of the final products (physical, textural and consumer acceptance) were investigated. Finally, the optimal process conditions required for creating a desirable final product were defined by applying the genetic algorithm (GA).

## 2. Materials and Methods

### 2.1. Materials

Commercial rice flour was provided by Hemija Commerc d.o.o. (Novi Sad, Serbia), while unroasted chicory root flour was procured from Patent Co. (Mišićevo, Serbia). Proximate compositions included total moisture (AOAC 925.10), ash (AOAC 923.03), protein (AOAC 979.09), total dietary fiber (AOAC 985.29), and carbohydrate contents (calculated by difference) (Table 1) of raw ingredients were determined according to procedures reported by Pasqualone et al. [16]. Total fat content was obtained by applying Weibull–Stoldt method [17]. Inulin content was determined according to Perović et al. [18]. All chemicals and reagents were obtained from official suppliers and were of analytical grade.

### 2.2. Instruments

The blends were mixed for 2 min in a twin-shaft paddle mixer that is part of the laboratory vacuum coater (model F-6-RVC, Forberg International AS, Norway). The extrusion was performed in a twin-screw extruder Bühler BTSK 30/28D (Bühler, Uzwil, Switzerland) with seven zones, a barrel length of 880 mm and length/diameter ratio = 28:1. The diameters of extrudates were measured with a movable beak gauge (MIB Messzeuge GmbH, Spangenberg, Germany). The bulk density was measured with apparatus from Tonindustrie (West und Goslar, Germany). The volume and density of extrudates were measured with Mettler Toledo Density Kit. Color parameters were measured using a Konica Minolta Chrome Meter colorimeter. Thermal characteristics were measured with the DSC 204 F1 Phoenix^®^ (Netzsch, Selb, Germany). Textural properties were measured with TA-XT2 Texture Analyzer (Stable Micro Systems, Surrey, UK).

### 2.3. Preparation of Blends for the Extrusion

Five different rice–CRF blends were prepared (containing 20, 24.1, 30, 35.9 or 40% of CRF). The control sample (CS) was prepared without CRF flour addition. The blends were mixed for 2 min and then transferred to the extruder.

### 2.4. Production of Novel Rice-Based CRF Snacks

Central composite design (CCD) was employed to generate 15 different rice snack formulations by varying feed moisture (M, 16.3–22.5%), screw speed (SS, 500–900 rpm) and CRF content (P, 20–40%) with six repetitions in central point (samples 2, 4, 9, 14, 19, and 20) (Table 2). CS was produced at 18% M and 800 rpm. These ranges were chosen according to the preliminary study (data not shown). The extruder barrel was jacketed, and water was used as a medium for the heating/cooling of the sections. The temperature along the barrel was controlled by two temperature control units (Regloplas P140 smart/RT61, Regloplas AG, St. Galen, Switzerland). The first unit-controlled temperature was set in sections 2, 3 and 4 (set at 60 °C), while a second one controlled the temperature in sections 6 and 7 (set at 120 °C). The diameter of the die opening was 4 mm (total open area 12.56 mm^2^), and the feed rate of dry blends was constant and set at 60 kg/h. The blends were moisturized directly in the extruder barrel, according to CCD. For this purpose, water was directly added at the end of the barrel′s first section via a cavity pump (model NM005BY, Netzsch, Waldkraiburg, Germany). The extrudates were allowed to cool down and dry until moisture content reached <10%, and then were packed in plastic containers till further analysis.

### 2.5. Extrudate Characterization

#### 2.5.1. Expansion Index (EI)

EI was determined in 10 replicates as the ratio of the extrudate and die diameters (n = 10), according to Vallée et al. [19].

#### 2.5.2. Bulk Density (BD)

BD of extrudates was calculated as the ratio of extrudate mass and cylinder volume [20] (Equation (1)):

(1) $$\mathrm{Bulk}\;\mathrm{density}=\frac{\left[\mathrm{Weight}\;\mathrm{of}\;\mathrm{extrudate}\right]\;\left(g\right)}{\left[\mathrm{Cylinder}\;\mathrm{volume}\right]\;\left(L\right)}$$

#### 2.5.3. Volume and Density

The volume and density of the extrudates were determined according to the manufacturer’s instructions manual from the Metller Toledo Density Kit [21].

The density of a single extrudate (D, kg/m^3^) was calculated according to Equation (2):

(2) $$D=\frac{A}{A-B}\cdot \left(\rho 0-\rho L\right)+\rho L$$

where A represents the mass of dry extrudate (g), B represents the mass of extrudate submerged in liquid (g), ρ0 refers to the density of the auxiliary liquid (g/cm^3^), while ρL is the density of air (0.0012 g/cm^3^).

The extrudates volume (V, m^3^) was determined according to the following Equation (3):

(3) $$V=\alpha \cdot \frac{A-B}{\rho 0-\rho L}$$

where *α* represents the weight correction factor (0.99985).

#### 2.5.4. Color Measurements

The color parameters were determined according to Vallée et al. (2017) [19] in five replications. The results were expressed as L* (lightness/darkness), a* (redness/greenness), b* (yellowness/blueness).

The total color difference (ΔE) between the control and the CRF-containing samples was calculated according to Ačkar et al. [22].

The whiteness index (WI) was determined according to study of Smarzyński et al. [23].

#### 2.5.5. Thermal Properties of Extrudates

The thermal characteristics of snack products were determined by differential scanning calorimetry according to the procedure of Wang et al. [24]. About 6 mg of the samples were weighed directly into aluminum pans so that the sample covered the bottom. Then distilled water (µL) was added so that the ratio of the sample to the added water was 1:3 (*w*/*v*). The pans are hermetically sealed and left to stand for an hour before analysis at room temperature. The calorimeter was calibrated using an indium standard, while an empty aluminum pan served as a reference sample. The samples were analyzed according to the method of Wang et al. [24] and the analysis conditions were as follows: the samples were tempered for 1 min at 20 °C, then heated from 20–120 °C at a heating rate of 10 °C/min, held for 1 min at 120 °C, and then cooled to room temperature (25 °C). The values of enthalpy change (ΔH, J/g) were recorded on that occasion.

#### 2.5.6. Textural Properties

The textural properties (hardness and crispiness index) of snack products were determined using a diametral compression test at room temperature (25 °C). Tests were performed by placing a single snack on a flat surface of the device and applying compression using a cylindrical stainless-steel probe with 45 mm diameter (P45) at a load cell of 50 kg and a breaking force of 5 g. The speed before, during, and after the measurement was 2.0 mmsec, 1 mm/s, 10 mm/s, respectively. The probe crossed the path required to achieve a sample deformation of 70%, while the threshold was set to 0.49. Texture Exponent software v.5.1.2.0 (Stable Micro Systems, UK) was used to obtain values from the force-deformation curve to calculate crispiness index (Ci) [25] and to determine hardness. The crispiness index is a measure of the crispiness of a product and is calculated according to Equation (4):

(4) $$\mathrm{Ci}=\frac{LL}{A\cdot \mathrm{Fmean}}$$

where *LL* is the normalized curve length (length of actual curve/Fmax), *A* is the area under the force-distance curve (Nxmm), and Fmean is the sum of the actual force values divided by the number of peaks (N) [26].

The hardness of the snack is defined as the force required to achieve the first fracture of the extrudate, read as the maximum value from the obtained curve of force to the probe path dependence (N) [27]. All textural properties were determined in 21 samples (20 rice-based samples enriched with chicory root flour and 1 control sample) within 30 replications.

#### 2.5.7. Sensory Analysis

Descriptive sensory analysis of seven selected extrudate samples (non-expanded samples were not used for sensory analysis similar to do Carmo et al. [1], EI ˃ 1.9) was conducted with a panel of trained sensory panelists with experience in the analysis of similar products (n = 8, 6 women and 2 men, aged 25 to 50 years). During training sessions, panelists agreed on the list of 18 sensory descriptors relating to the samples’ appearance, odor, taste, texture, and residual sensations. The intensity of the observed sensory properties was evaluated on a continuous linear scale of 100 mm anchored with appropriate words (Supplementary Table S1). The samples were coded with three randomly chosen digit numbers and delivered individually, one by one. The evaluation was conducted in two subsequent sessions in separate air-conditioned (22 °C) sensory evaluation booths. Distilled water and slices of apple were provided to cleanse the palate. All participants received written information about the sensory study, and they signed informed consent to participate. The sensory study was approved by the Ethics Committee of the Institute of Food Technology in Novi Sad, University of Novi Sad, Serbia (Ref. No. 175/I/16-3 from 8 February 2022).

### 2.6. Statistical Analysis

All experiments were performed in designated repetitions, and the results are presented as means ± SD.

The Principal Component Analysis (PCA) was performed on the matrix of Pearson correlation coefficients. Statistical data processing was carried out using the statistical package XLSTAT 2020.5.1

The artificial neural network (ANN) was employed to investigate the impact of extrusion process variables (M, SS, and P) on output variables (EI, BD, D, V, L*, a*, b*, ΔE, DG, Ci and Hardness). Input and output data were normalized (min-max normalization method) prior ANN calculation [28]. The training process of the network (in the form of Multilayer Perceptron-MLP) using 20 data points (every point refers to one sample) was repeated 100,000 times, changing the various ANN topologies, with a different number of neurons in the hidden and the output layers (5–20), different activation functions, using random initial values of weight coefficients and biases. The optimization of the ANN structure was performed by minimizing the validation error, and generated ANN predicates 11 output parameters simultaneously. The Broyden–Fletcher–Goldfarb–Shanno algorithm (BFGS) was used during the ANN modelling, according to Voca et al. [28], to expedite the computation and maintain the solution’s convergence. The performance of created ANN models was tested through the R^2^ value (coefficient of determination).

## 3. Results

### 3.1. Extrusion Processing–Experimental Design

**Figure 1:**
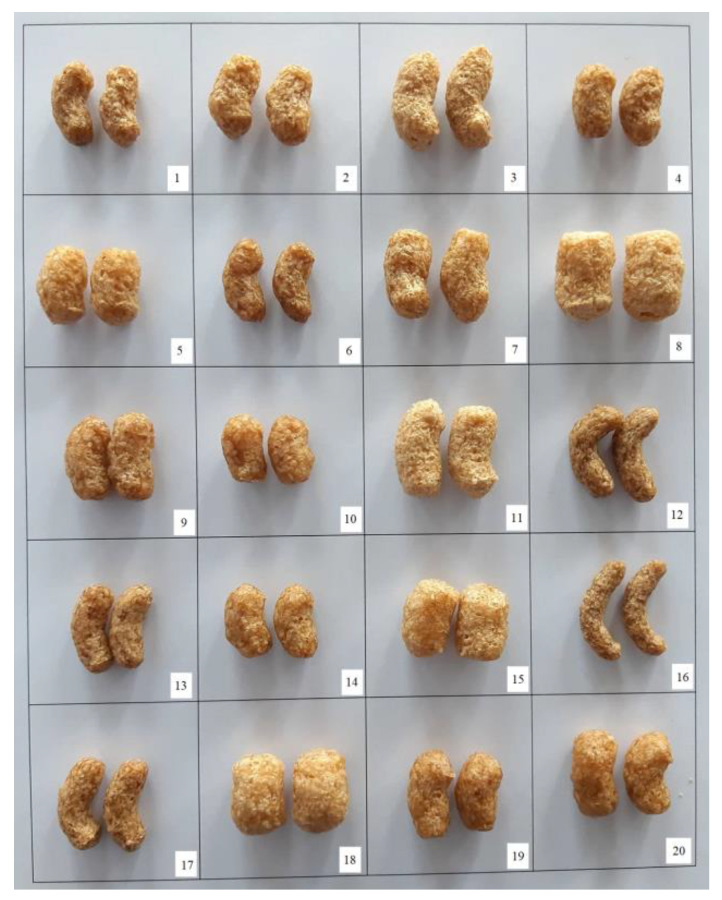
Rice-based chicory root enriched extrudates obtained following CCD. Sample numbers (1–20) refer to different extrusion conditions defined by CCD obtained through varying moisture content (16.3–22.5%), screw speed (500–900 rpm), and chicory root content (20–40%).

Following the CCD, twenty extrudates were obtained under different extrusion conditions (Figure 1) and the dependent variables are presented in Table 2 (EI, BD, D, V, color parameters, DG, Ci, and hardness). Six central points (samples 2, 4, 9, 14, 19 and 20) were produced to check the repeatability of the extrusion process expressed through the coefficient of variation (CV). All calculated CVs (Table 2) were lower than 10%, which confirmed the adequacy of the applied measurement.

### 3.2. Process Conditions Effect on Physicochemical Properties

**Figure 2:**
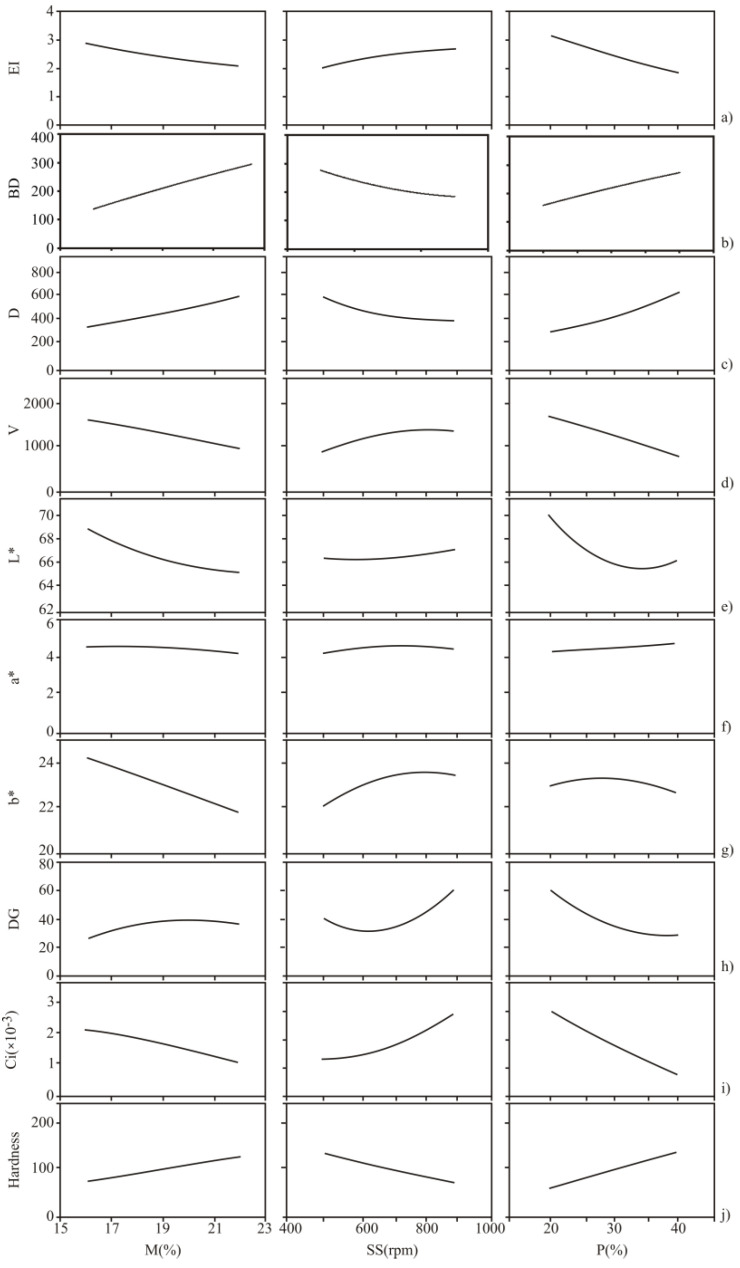
Effect of extrusion process parameters (M—moisture content, %; SS—screw speed, rpm; P—chicory root flour content, %) on investigated parameters (EI—expansion index, BD—bulk density, D—extrudate′s density, V—extrudates′s volume, L*—extrudates′s lightness, a*—extrudate′s redness/greenness, b*—extrudate′s yellowness/blueness, DG—degree od gelatinization, Ci—crispiness index) (**a**–**j**) by ANN.

Figure 2 shows the effect of extrusion process variables on investigated physicochemical properties.

#### 3.2.1. Expansion Index

High values of expansion and low values of density are desirable characteristics of extrudates that depend on many process conditions [29].

An increase in starting blends’ moisture content had a negative effect on EI (Figure 2a), which was likely due to the reduced strain force and generated lower pressure at the die exit. Actually, the high moisture content dilutes the material in the barrel, which allows for its easier and faster flow and shortens the time necessary for the extrusion cooking of material. This occurrence inhibited the creation of a larger number of pores and thus reduced product expansion. A Similar trend was noted in the study of Seth et al. [30].

Screw speed adjustment is a powerful tool for obtaining the desirable expansion of the final product. Screw speed enhancement increases the EI of obtained snacks (Figure 2a). This trend may be explained by the gradual increase of shear forces inside the extruder barrel responsible for the material’s macromolecular degradation, resulting in the uniform dough, which consequently obtains better expansion properties when coming out of the die. A similar observation was made by Zhao et al. [31].

The addition of CRF led to a decrease in EI (Figure 2a), possibly due to fibers (inulin) from chicory root [32]. Fibers reduce the amount of available water necessary for expansion by binding it. Furthermore, fibers are not compatible with starch and interfere with the formation of starch film around the air cells preventing their growth. Fibers also reduce starch elastic properties and swelling, generating low product expansion. Nascimento et al. [33] noted the same trend.

CS showed the highest EI (4.84) due to the absence of fibers. The obtained results of CRF enriched blends showed a rise in dietary fiber inulin with an increase in CRF share, while there was a noted absence of inulin in rice flour and CS (Table 3). Namely, starch is a basic component necessary for a large expansion of the product, while other additives (proteins, fibers, sugars, fats) act as diluents [34]. Despite this, optimal sample 8 also had good expansion (3.34) and the highest EI after CS.

#### 3.2.2. Bulk Density

Bulk density (BD) is an important parameter of extruded products and is significant for designing packaging requirements. BD is determined by the product’s density, shape and size [35].

The moisture rise increases the BD (Figure 2j) of the rice-CRF extrudates. Namely, the high moisture content may linger in the extruder barrel, resulting in a higher pressure differential at the die end. This possibly disables expansion and generates more compact products. A well-known fact that EI and BD are negatively correlated is consistent with our study (with a correlation factor equal to −0.912, statistically significant at *p* < 0.001 level), as well as with the observations of Kojić et al. [36].

A rise in screw speed resulted in extrudates with lower BD of rice based CRF extrudates. In fact, the high screw speed increases process energy input (Supplementary Table S2). Consequently, it accelerates a high sudden pressure drop at the die, resulting in less dense extrudates, consistent with the study of Alam et al. [37].

The fibers from CRF are responsible for increased BD due to its impact on dough’s flow behavior and formulation of compact product structure. A similar trend was reported after the addition of carrot pomace and cauliflower trimming powders rich in fibers into the soybean-rice extrudates [37]. Inulin from CRF is a dietary probiotic fiber, which may be used as a thickening agent. Long-chain inulin, together with starch, has the ability to form high-hardness gels and consequently cause the high bulk density in the product [38].

The CS showed the lowest BD (65.9 g/L), indicating that replacement of part of the rice starch with CRF rich in sugars and fibers resulted in extrudates with lower expansion, i.e., higher BD. The same trend was observed for rice extrudate in the study by El-Samahy et al. [39], who added cactus puree (5–20%). Although adding such raw materials may slightly impair the product’s physical properties, as it was the case with the optimal sample (110.33 g/L), the newly formed extrudates may have improved functional, nutritional, and sensory properties.

#### 3.2.3. Density and Volume of Extrudates

The extrudate density ranged from 250–830 kg/m^3^, while the extrudate volumes were 640–2040 m^3^. The highest density was recorded for sample 16, which had the lowest expansion (1.44 kg/m^3^), while sample 8 had the lowest density and the highest expansion (3.34 kg/m^3^). CS resulted in 110 kg/m^3^ for D and 3100 m^3^ for V, while optimal sample 8 had 250 kg/m^3^ for D and 2040 m^3^ for V (Table 2).

A lower value of D is a desirable characteristic of extrudates and is inversely proportional to EI. A negative correlation between D and EI was observed within this study, with a correlation factor equal to −0.895, statistically significant at *p* < 0.001 level. Extrusion cooking promotes starch gelatinization, increasing the product volume while reducing its density. It was reported that the addition of various raw materials, mainly of plant origin, causes changes in the density of extrudates [29].

The increase in the feed moisture content increased extrudate density and reduced extrudate volume (Figure 2c,d). Higher moisture content reduces the melting of starch granules due to the reduction of adhesion of the mixture inside the barrel, affecting negatively starch gelatinization, thus decreasing expansion and increasing apparent density [32]. Tsokolar-Tsikopoulos et al. [32] reported the same trend during the production of extrudates with the addition of inulin, where moisture probably affects the changes in the molecular structure of amylopectin from starch materials. Moisture may affect bubble walls to become softened and contract and produce less expanded products with poor volume [40], which is also presented in our results (Figure 2a,d).

An increase in the screw speed resulted in the study’s extrudates with lower D and higher V. High screw speeds diminished melt viscosity, accelerated bubble growth, and resulted in puffier products with high volume (Figure 2c,d). Such conclusions were also derived by Yağci et al. [41].

The addition of CRF caused an increase in density and a decrease in volume (Figure 2c,d), which was likely due to the presence of inulin (Table 3), as it was also reported by Hegazy et al. [42]. They noticed an increase in fiber content after adding the chickpea and tomatoes compared to the CS. Raw materials rich in fiber, such as CRF, contribute the most to the increase in product density because they affect the capability of “packaging” molecules. Linear polymers such as dietary fibers (inulin) are capable of packing more efficiently.

#### 3.2.4. Color Properties

The lightness (L*) of the samples ranged from 63.16 to 70.48, redness (a*) showed values from 3.57 to 5.17, while yellowness (b*) was recorded in the range of 20.52 to 24.49. The CS sample showed the highest lightness (L* = 99.94), slight green (a* = –1.24) and bright yellow (b* = 8.62) nuances. The total color difference, i.e., the difference in raw materials and final product color, ranged from 11.64 to 18.03. WI ranged from 56.46 (for sample 12 with the highest share of CRF, i.e., 40%) to 91.41 for the control sample (Table 2). The addition of CRF led to increase in ΔE and a decrease in WI due to the darker color characteristics of this raw material (L* = 63.75, a* = 4.33, b* = 17.23). Furthermore, the presence of the fiber inulin in CRF, which determines the extent of the non-enzymatic browning reaction, may be responsible for decrease in L* and an increase in a * and b * values indicating a high rate of Maillard′s reactions compared to CS, as it was also noted in the study of Morris and Morris [43] and Rayan et al. [44].

The rise in moisture content caused a decrease in L*, a* and b* (Figure 2e–g). Moisture may affect biochemical transformations, such as Maillard’s reactions, which causes a reduction of the investigated color parameters [45]. The increase in moisture causes a decrease in L* and thus gives higher values of ΔE [46]. The highest color change (18.03) was observed in the sample with the highest moisture content (22.5%) (Figure 2e).

Increasing the screw speed increased the values for L*, a* and b*. These results can be explained by the shorter retention of the raw material in the extruder barrel where the time of pigment degradation is reduced, i.e., the time required to form the darker color of the extrudate (Figure 2e,f,h). Similar conclusions were noted by Kaur et al. [47].

On the other hand, the increase in CRF content resulted in noticeable changes in ΔE (samples 12 and 18), giving the darker color of extrudates, as noted in the study of Rayan et al. [44]. The products obtained within this study manifested attractive color properties (reduced light (L*), red (a*) and blue (b*) color attributes) caused by Maillard′s tanning reactions (Figure 2e–g). The same observations were reported in the study of Dalbhagat et al. [45].

#### 3.2.5. Thermal Properties

It is important to determine the impact of extrusion process conditions on starch-based extrudate thermal properties due to its relationship with digestion. The higher the degree of gelatinization, the easier the snack product is to digest [24].

The enthalpy value of non-extruded mixtures decreased with the addition of CRF (from 4.50 to 2.57 J/g, and from 3.12 to 0.68 J/g for the second peak, Supplementary Table S3) and was caused by decreasing the share of available starch necessary for gelatinization. This is in accordance with the results of Wang et al. [16], where the enthalpy value decreased with adding rice bran to rice extrudates. Furthermore, some unstable starch crystals may be transformed into more stable starch crystallites, probably due to amylose and amylopectin interaction in the amorphous lamellae giving lower ΔH [48]. This occurred probably due to interaction between more stable starch and inulin functional groups, which resulted in extension of amorphous areas in the starch [49].

The appearance of the second endothermic peak in non-extruded mixtures with the addition of chicory root in the temperature region of 70.8–82.6 °C (Supplementary Table S3) can be considered as the dissociation temperature of the lipid-amylose complex, which was previously found to occur in the temperature range of 70–108 °C [24].

The DG ranged from 14.18% to 69.70%. DGs for CS and optimal sample (no. 8) were 46.21% and 69.70% (Table 2), respectively, highlighting the optimal extrudate as more digestible. The enthalpy of gelatinization of snack products ranged from 1.24–3.57 J/g, or 1.38 J/g for the CS (Supplementary Table S4).

The increase in moisture positively impacted DG by allowing starch molecules to move freely, while heat penetrated more easily into the material and thus accelerated gelatinization. Furthermore, the high moisture content may reduce the elasticity of dough due to plasticization of the melt which increases DG. This trend was also noted in the study of Samyor et al. [50].

The initial increase in the screw speed (from 500 to 600 rpm) slightly reduces DG probably due to its impact on lowering starting viscosity of the melt. A further rise in screw speed (over 600 rpm) led to an increase in DG, which may be explained by intensive mixing in the barrel and uniform heating of the dough and enhanced starch gelatinization (Figure 2h). Similar conclusions were also found in the investigations of Kaur et al. (2014) [47].

CRF addition lowered DG, because dietary fibers from chicory root probably limited the available water as well as the processability of rice starch, thus reducing its enlargement and gelatinization (Figure 2h).

#### 3.2.6. Textural Properties

##### Crispiness Index

The high crispiness is accompanied by high Ci values [25]. The obtained results suggest that Ci values depend on both sample formulation and the processing parameters. CS showed the highest crispiness index (6.47 × 10^−3^), while by increasing CRF content Ci values decreased (Figure 2i). The values of the Ci for CRF-containing samples ranged from 0.09 to 3.25 × 10^−3^ (Table 2). Fibers present in CRF may affect cellular structure of extrudates, reducing product’s expansion and crispiness while enhancing density and hardness [25]. The fiber enrichment brings dilution of starch, impulsive air cell rupture, and thinning of the cell wall. A similar finding was reported by Nascimento et al. [33], who investigated the influence of the addition of fiber on the Ci.

The moisture content and screw speed showed an inverse impact on Ci of rice-CRF extrudates (Figure 2i). Higher moisture content hinders the formation of a puffy structure, generates thick structures and forms less crispy extrudates. On the other hand, higher screw speed causes an increase in melt temperature producing expanded and crispy extrudates. These findings are in agreement with da Silva et al. [51]. Extrudates produced at the highest moisture content (22.5%) and at the lowest screw speed (500 rpm) showed low values of the Ci (1.29 × 10^−3^ and 0.90 × 10^−3^, respectively), low values of EI (2.19 and 2.02) and higher values of density (520.00 and 550.00 kg/m^3^) (Table 2).

##### Hardness

Hardness of the extruded snack products is one of the most critical factors affecting consumer acceptability and is closely related to extrudates expansion and cellular structure [27]. Similar to the crispiness index, the results showed that samples hardness was highly influenced by formulation and processing parameters. Namely, an increase in the moisture led to an increase in sample hardness (Figure 2j). Water acts as a plasticizer, reduces starch viscosity and mechanical energy, and disables the formulation of porous structures, resulting in firm products [31]. Furthermore, an increase of CRF in sample formulation causes an increase in sample hardness due to the presence of fibers that could not be incorporated into internal pores during extrusion, producing thick-packed structures followed by an increase in hardness [31]. The CRF fibers may provide extrudates structural integrity due to the interaction with proteins, resulting in a denser product, similar to the work of Poliszko et al. [52]. The hardness of CRF-containing samples ranged from 61.33 to 237.29 N (Table 2) compared to the CS, which showed a hardness of 43.48 N. The hardness of the sample extruded under optimal conditions (Sample 8) was 98.74 N indicating that CRF addition of a 24.1% does not greatly impair the hardness of the product compared to the CS. Moreover, a negative correlation was noticed between Ci and hardness (r = −0.70, *p* < 0.001).

The rise in screw speed resulted in products of low hardness (Figure 2j). This phenomenon probably occurs due to the generation of material pushing force to the outlet during fast screw rotation, which enables better expansion of extrudates and results in light and less rough products. The same trend was noted by Tsokolar-Tsikopoulos et al. [32].

#### 3.2.7. Sensory Analysis

The sensory profile of samples was defined based on appearance, odor, taste, flavor, texture, and residual impressions (Table 4).

The results are given in arithmetic means and standard deviations (*n* = 16, 8 assessors in two separate sessions). The values in the same row marked with different letters differed statistically significantly (*p* < 0.05) using the Tukey HSD test. The bold F-values indicate that there is no statistically significant difference (*p* > 0.05) between the samples in terms of the observed property. TO—Total odor intensity, RO—The intensity of the odor of rice, FRO—Odor intensity of fried rice, CO—The intensity of the odor of chicory, BT—Bitterness, BT2—Bitter taste after 2 min, TF—Total flavor intensity, CF—Flavor intensity of chicory.

**Figure 3:**
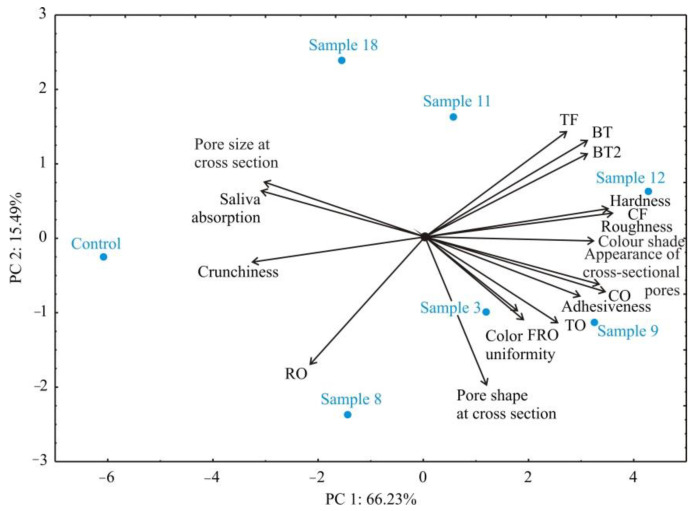
PCA biplot diagram for analyzed samples and observed sensory properties (TO—Total odor intensity, RO—The intensity of the odor on rice, FRO—Odor intensity on fried rice, CO—The intensity of the odor on chicory, BT—Bitterness, BT2—Bitter taste after 2 min, TF—Total flavor intensity, CF—Flavor intensity on chicory).

In order to better understand correlations among samples and correlations between samples and analyzed sensory properties, the Principal Component Analysis (PCA) was applied (Figure 3).

CS is clearly distinguished from the other analyzed samples primarily due to pronounced crunchiness and saliva absorption during mastication. Furthermore, it can be characterized by white color, very mild flavor, and odor typical for cooked rice of moderate intensity. The CRF-containing samples (particularly Sample 9 and Sample 12) were perceived as significantly darker and more bitter with highly pronounced overall chicory odor and flavor in comparison to the CS. Bitterness perceived in samples containing CRF can be connected with the presence of sesquiterpene lactones [7], which are reported to possess a bitter taste. The most profound and persistent bitter taste was perceived in Sample 12, containing 40% of CRF. The sample produced under optimal conditions (Sample 8) was significantly less bitter, with a less profound odor and flavor that resembled chicory compared to the majority of the CRF-containing samples.

The observed differences in textural properties of analyzed samples come primarily from the CRF content in sample formulation. Namely, samples 9, 12 and 18 possess different CRF content (30, 40 and 20%, respectively) but were processed under the same processing conditions and the perceived hardness, roughness and adhesiveness were significantly (*p* < 0.05) different. Samples processed at higher moisture content and lower screw speed (Samples 9 and 12—right side of the map) were associated with negative sensory attributes for this type of products, such as feeling hard, rough and adhesive during chewing, with waxy appearance of the cavities and with an intense and persistent bitter taste and intense odor and flavor resembling of chicory. On the other hand, The control sample and Sample 8, processed at lower moisture content and higher screw speed (positioned on the left side and middle way on the map, respectively), were described as crunchy and with moderately large cavities, attributes positively associated with this kind of products. Similar findings were reported by do Carmo et al. [1], who investigated the influence of extrusion parameters on sensorial properties of expanded snacks from pea and oat fractions.

### 3.3. Optimization of ANN Model

The developed ANN model demonstrated very good generalization capability and adequate experimental data prediction. The optimal number of neurons in the hidden layer for EI, BD, D, V, L*, a*, b*, ΔE, DG, Ci and Hardness prediction was 10 (network MLP 3-10-11) to obtain high values of R^2^ (overall 0.999 for the training period) and the low sum of squares values (Supplementary Table S5).

The quality of the ANN model fit was tested in Supplementary Table S6 where the numerical verification of the created ANN model was tested using the commonly applied statistical parameters. Lower values of reduced chi-square (χ^2^), mean bias error (MBE), root mean square error (RMSE) and mean percentage error (MPE), the sum of squared errors (SSE) and average absolute relative deviation (AARD), as well as the R^2^ value close to 1 were indicative that the ANN model was adequate for output parameters prediction. This means that the ANN model developed for each of investigated outputs should provide accurate results when cycling through the algorithm routine for the next optimization step. The “goodness of fit” tests parameters for the developed ANN model (Supplementary Table S6) were calculated according to Voca et al. [28] and showed good prediction of ANN model.

### 3.4. Genetic Algorithm (GA) Modeling for Obtaining Optimal Process Conditions

The developed ANN model was used with an idea to find the process factors of the gluten-free snack enriched with CRF production that will result in the maximal EI, V, DG and Ci, and the minimal BD, D and Hardness values using the genetic algorithm (GA). The constraints used in the optimization procedure were applied within the experimental range of parameters. The initial population was randomly generated, while the next generations were calculated by distance measure and non-dominated ranking of the individual points in the present generation [14].

The number of generations reached 412 for ANN, while the size of the population was set to 100 for each input variable. The calculated maximum values of EI, V, DG and Ci during the process were: 3.34, 2040 m^3^, 69.7% and 3.33 × 10^−3^, respectively, while the calculated minimum values of BD, D and Hardness were: 110.33 g/L, 2.50 kg/m^3^ and 98.74 N, respectively. The optimal process conditions necessary for obtaining desirable physical and textural properties of extrudate generated by GA were defined at: M = 17.6%, SS = 820 rpm and P = 24.1% (Sample 8).

## 4. Conclusions

This study describes the optimization process for the design of novel gluten-free rice extrudates enriched with chicory root flour to expand the snack market supply while satisfying consumers’ demands.

Moisture and CRF contents showed a higher impact on the measured parameters when compared to the screw speed. The desirable extrusion conditions for the production of acceptable extrudates were defined by a genetic algorithm (moisture content 17.6%, screw speed 820 rpm and CRF content 24.1%). Meanwhile, the addition of CRF may potentially contribute to the improved functional aspects of obtained snacks, optimal process conditions can provide a product with acceptable physicochemical properties, as well as the development of an attractive product color appearance compared to the control sample.

Considering that CRF may contribute to the nutritional and functional profile of the snack products, the increase of knowledge on the extrusion conditions’ influence on the physicochemical and sensory properties of extrudates enriched in CRF will be applicable. In addition, further studies will be devoted to the investigation of CRF addition effect on the functional aspect of extrudates.

## Figures and Tables

**Table 1:** Proximate composition of raw ingredients.

Material	Moisture, %	Ash, %	Protein, %	Fat, %	Total Dietary Fiber, %	Total Carbohydrates, %
Chicory root flour	6.72	3.59	6.28	0.39	42.41	40.61
Rice flour	13.30	0.67	7.0	0.69	1.32	77.02

**Table 2:** CCD design and measured dependent process variables (EI, BD, D, V, color properties), measured thermal (DG) and textural (Ci, Hardness) properties.

	CCD Design				Measured Variables										
S. No.	M %	SS Rpm	P %	EI	BD g/L	D kg/m^3^	V m^3^	L*	a*	b*	ΔE	WI	DG %	Ci × 10^−3^	Hardness N
1	21.2	820	35.9	2.15 ± 0.34 ^ab^	272 ± 2.86 ^g^	540 ± 0.62 ^l^	980 ± 1.03 ^ab^	65.19 ± 0.99 ^bc^	4.28 ± 0.21 ^bcdef^	22.36 ± 0.40 ^cde^	14.97 ± 1.11 ^f^	58.53 ± 0.16 ^gf^	51.09 ± 1.23 ^de^	1.08 ± 0.00 ^e^	110.36 ± 23.90 ^b^
2	19.4	700	30.0	2.50 ± 0.43 ^ab^	212 ± 1.26 ^fg^	418 ± 0.12 ^h^	1340 ± 0.65 ^bc^	64.10 ± 1.24 ^ab^	5.10 ± 0.31 ^efg^	23.23 ± 0.75 ^cdefg^	17.88 ± 1.23 ^k^	56.82 ± 0.01 ^i^	22.82 ± 0.88 ^b^	1.68 ± 0.00 ^h^	103.24 ± 15.75 ^b^
3	17.6	820	35.9	2.45 ± 0.56 ^ab^	187 ± 0.98 ^e^	540 ± 0.55 ^l^	960 ± 0.22 ^ab^	70.06 ± 0.42 ^g^	4.27 ± 0.16 ^bcdef^	23.80 ± 0.58 ^fgh^	11.64 ± 1.03 ^b^	61.64 ± 0.12 ^c^	43.21 ± 0.87 ^c^	1.51 ± 0.00 ^g^	88.82 ± 17.49 ^b^
4	19.4	700	30.0	2.47 ± 0.44 ^ab^	218 ± 2.46 ^fg^	419 ± 0.22 ^h^	1350 ± 0.90 ^bc^	63.78 ± 1.26 ^ab^	5.09 ± 0.28 ^efg^	23.25 ± 0.52 ^cdefg^	17.83 ± 1.87 ^k^	56.81 ± 0.02 ^i^	23.84 ± 0.16 ^b^	1.66 ± 0.00 ^h^	104.94 ± 16.54 ^b^
5	21.2	820	24.1	2.64 ± 0.54 ^ab^	211 ± 2.07 ^d^	360 ± 0.26 ^g^	1400 ± 0.93 ^bc^	67.55 ± 0.50 ^ef^	4.09 ± 0.22 ^bcd^	22.15 ± 0.76 ^cd^	15.02 ± 1.34 ^f^	60.62 ± 0.11 ^d^	56.96 ± 1.09 ^efg^	2.16 ± 0.00 ^j^	72.06 ± 14.81 ^b^
6	22.5	700	30.0	2.19 ± 0.42 ^ab^	283 ± 2.54 ^k^	520 ± 0.30 ^k^	1020 ± 0.91 ^ab^	63.16 ± 1.77 ^a^	4.84 ± 0.28 ^efg^	22.34 ± 0.78 ^cde^	18.03 ± 1.35 ^k^	56.76 ± 0.21 ^ji^	60.96 ± 0.92 ^g^	1.29 ± 0.00 ^f^	118.64 ± 26.92 ^b^
7	19.4	900	30.0	2.71 ± 0.43 ^ab^	183 ± 0.30 ^e^	350 ± 0.14 ^f^	1520 ± 0.22 ^bc^	65.17 ± 0.57 ^bc^	4.91 ± 0.17 ^efg^	23.58 ± 0.33 ^efgh^	16.81 ± 1.63 ^j^	57.78 ± 0.17 ^h^	44.64 ± 0.53 ^c^	2.25 ± 0.00 ^n^	66.45 ± 11.91 ^b^
8	17.6	820	24.1	3.34 ± 0.46 ^b^	110 ± 0.68 ^b^	250 ± 0.17 ^c^	2040 ± 0.41 ^bc^	68.54 ± 0.53 ^fg^	4.58 ± 0.13 ^defg^	24.49 ± 0.26 ^h^	15.55 ± 1.31 ^k^	59.99 ± 0.14 ^e^	69.70 ± 2.43 ^h^	3.22 ± 0.00 ^k^	98.74 ± 18.11 ^b^
9	19.4	700	30.0	2.49 ± 0.28 ^ab^	223 ± 1.66 ^fg^	419 ± 0.26 ^h^	1345 ± 0.96 ^bc^	64.08 ± 0.89 ^ab^	5.09 ± 0.30 ^efg^	23.46 ± 0.46 ^cdefg^	17.87 ± 1.29 ^g^	56.81 ± 0.09 ^i^	22.91 ± 0.63 ^b^	1.68 ± 0.00 ^h^	113.68 ± 20.62 ^b^
10	21.2	580	24.1	2.40 ± 0.39 ^ab^	267 ± 2.14 ^ij^	500 ± 0.24 ^j^	1080 ± 0.70 ^ab^	66.53 ± 0.95 ^cde^	4.37 ± 0.22 ^cdef^	22.49 ± 0.39 ^cdef^	16.11 ± 1.02 ^h^	59.56 ± 0.18 ^e^	24.70 ± 1.34 ^b^	0.93 ± 0.00 ^cd^	102.25 ± 21.32 ^b^
11	16.3	700	30.0	2.84 ± 0.36 ^ab^	138 ± 1.59 ^c^	310 ± 0.15 ^d^	1730 ± 0.10 ^c^	68.01 ± 0.47 ^ef^	4.82 ± 0.16 ^efg^	24.31 ± 0.34 ^gh^	14.87 ± 0.98 ^f^	59.65 ± 0.13 ^e^	41.32 ± 0.32 ^c^	2.05 ± 0.00 ^i^	68.91 ± 15.70 ^b^
12	19.4	700	40.0	1.94 ± 0.28 ^ab^	261 ± 2.20 ^i^	560 ± 0.28 ^n^	990 ± 0.93 ^ab^	63.32 ± 0.74 ^ab^	5.10 ± 0.25 ^g^	23.11 ± 0.42 ^cdefgh^	16.42 ± 1.26 ^g^	56.21 ± 0.09 ^j^	14.43 ± 0.62 ^a^	0.74 ± 0.00 ^b^	125.00 ± 20.13 ^bc^
13	19.4	500	30.0	2.02 ± 0.17 ^ab^	273 ± 2.85 ^k^	550 ± 0.22 ^m^	970 ± 0.70 ^ab^	65.27 ± 0.64 ^bc^	4.46 ± 0.18 ^cdefg^	21.92 ± 0.39 ^bc^	15.89 ± 1.15 ^h^	58.81 ± 0.16 ^f^	43.18 ± 1.29 ^c^	0.90 ± 0.00 ^c^	126.50 ± 12.34 ^bc^
14	19.4	700	30.0	2.47 ± 0.28 ^ab^	213 ± 1.30 ^fg^	418 ± 0.28 ^h^	1355 ± 0.85 ^bc^	64.10 ± 0.53 ^ab^	5.10 ± 0.22 ^efg^	23.29 ± 0.42 ^cdefg^	17.93 ± 1.47 ^k^	56.80 ± 0.03 ^i^	22.88 ± 0.15 ^b^	1.66 ± 0.00 ^h^	103.21 ± 18.63 ^b^
15	17.6	580	24.1	2.90 ± 0.35 ^ab^	160 ± 1.43 ^d^	330 ± 0.16 ^e^	1570 ± 0.95 ^bc^	69.29 ± 0.91 ^fg^	4.23 ± 0.27 ^bcde^	23.09 ± 0.59 ^cdefgh^	14.07 ± 1.05 ^e^	61.46 ± 0.19 ^c^	54.50 ± 0.89 ^ef^	2.85 ± 0.00 ^l^	71.40 ± 11.62 ^b^
16	21.2	580	35.9	1.44 ± 0.36 ^a^	334 ± 1.74 ^l^	830 ± 0.46 ^o^	640 ± 0.35 ^a^	67.32 ± 0.41 ^def^	3.57 ± 0.07 ^b^	20.52 ± 0.43 ^b^	12.23 ± 0.87 ^c^	61.37 ± 0.22 ^c^	14.18 ± 1.43 ^a^	0.09 ± 0.00 ^a^	237.29 ± 37.91 ^c^
17	17.6	580	35.9	2.06 ± 0.21 ^ab^	243 ± 1.17 ^h^	470 ± 0.36 ^i^	990 ± 0.61 ^ab^	65.32 ± 0.79 ^bcd^	4.72 ± 0.14 ^defg^	23.37 ± 0.26 ^defgh^	15.37 ± 1.11 ^g^	58.04 ± 0.11 ^hg^	47.28 ± 2.33 ^cd^	0.94 ± 0.00 ^d^	129.71 ± 25.61 ^bc^
18	19.4	700	20.0	3.17 ± 0.25 ^ab^	159 ± 1.11 ^d^	310 ± 0.23 ^d^	1810 ± 0.49 ^c^	70.48 ± 0.67 ^g^	3.75 ± 0.16 ^bc^	22.69 ± 0.29 ^cdef^	12.95 ± 1.07 ^d^	62.70 ± 0.16 ^b^	59.61 ± 0.84 ^fg^	2.89 ± 0.00 ^m^	61.33 ± 10.46 ^b^
19	19.4	700	30.0	2.46 ± 0.53 ^ab^	217 ± 0.20 ^fg^	419 ± 0.12 ^h^	1340 ± 0.80 ^bc^	63.91 ± 0.65 ^ab^	5.09 ± 0.19 ^efg^	23.47 ± 0.41 ^cdefg^	17.87 ± 1.57 ^k^	56.80 ± 0.21 ^i^	22.75 ± 1.12 ^b^	1.67 ± 0.00 ^h^	114.95 ± 19.65 ^b^
20	19.4	700	30.0	2.47 ± 0.41 ^ab^	218 ± 2.87 ^fg^	418 ± 0.12 ^h^	1350 ± 0.79 ^bc^	64.62 ± 0.92 ^ab^	5.10 ± 0.36 ^efg^	23.45 ± 0.52 ^cdefg^	17.92 ± 1.72 ^k^	56.82 ± 0.14 ^i^	22.49 ± 0.71 ^b^	1.68 ± 0.00 ^h^	100.83 ± 19.08 ^b^
CS	16.0	800	00.0	4.84 ± 0.22 ^c^	66 ± 1.67 ^a^	110 ± 0.02 ^a^	3100 ± 0.04 ^d^	99.94 ± 0.37 ^h^	−1.24 ± 0.06 ^a^	8.62 ± 0.18 ^a^	1.23 ± 0.08 ^a^	91.41 ± 0.16 ^a^	46.21 ± 0.76 ^cd^	6.47 ± 0.01 ^o^	43.48 ± 2.21 ^a^
CV				4.55	2.53	4.08	0.61	1.83	4.84	2.09	4.88	0.02	1.64	0.48	5.08

S. no.—Sample number; M—moisture content, SS—screw speed, P—chicory root flour content, CS—control sample, EI—expansion index, BD—bulk density, D—density, V—volume, L*—lightness, a*—redness/greenness, b*—yellowness/blueness, ΔE—total color difference, WI—whiteness index, DG—degree of gelatinization, Ci—crispiness index, CV—coefficient of variation for six central points (samples 2, 4, 9, 14, 19 and 20). Values in the same column marked with different letters were statistically significantly (*p* < 0.05) different (Tukey HSD test).

**Table 3:** Inulin content (%) in raw materials, starting blends and control sample.

Sample	Inulin Content, %
Chicory root	30.65 ± 1.28
Rice flour	n.d.
Control sample	n.d.
20% CRF-rice blend	6.34 ± 0.11
24.1% CRF-rice blend	6.50 ± 0.51
30% CRF-rice blend	9.07 ± 0.05
35.9% CRF-rice blend	10.08 ± 0.30
40% CRF-rice blend	11.28 ± 0.32

n.d.—not detected.

**Table 4:** Sensory analysis of extrudate samples.

Samples								
Parameter	Control	Sample 3	Sample 8	Sample 9	Sample 11	Sample 12	Sample 18	F
Colour shade	0.0 ^d^	78.9 ± 10.7 ^b^	57.4 ± 11.1 ^c^	89.6 ± 8.3 ^ab^	61.5 ± 12.8 ^c^	95.3 ± 6.5 ^a^	60.6 ± 15.7 ^c^	73.7
Color uniformity	4.3 ± 10.5 ^b^	13.3 ± 9.5 ^b^	20.5 ± 16.8 ^ab^	39.9 ± 21.2 ^a^	10.8 ± 9.9 ^b^	12.1 ± 2.7 ^b^	13.4 ± 15.4 ^b^	5.7
Pore shape at cross-section	28.0 ± 17.2 ^a^	44.0 ± 31.2 ^a^	32.5 ± 29.6 ^a^	40.4 ± 26.5 ^a^	22.9 ± 17.1 ^a^	27.3 ± 35.7 ^a^	20.1 ± 16.9 ^a^	0.9
Appearance of cross-sectional pores	7.0 ± 4.6 ^c^	46.4 ± 19.9 ^ab^	23.5 ± 8.8 ^bc^	42.4 ± 23.2 ^ab^	29.3 ± 15.5 ^abc^	52.3 ± 28.0 ^a^	10.9 ± 6.4 ^c^	8.3
Pore size at cross-section	66.9 ± 13.9 ^a^	11.3 ± 4.7 ^d^	52.5 ± 6.3 ^bc^	12.9 ± 4.5 ^d^	45.5 ± 4.9 ^c^	22.0 ± 6.9 ^d^	60.1 ± 6.1 ^ab^	77.6
TO	4.9 ± 3.5 ^f^	20.5 ± 5.4 ^d^	43.1 ± 6.8 ^b^	34.3 ± 7.2 ^c^	15.8 ± 3.9 ^de^	54.0 ± 6.7 ^a^	7.9 ± 3.0 ^ef^	91.7
RO	16.0 ± 21.5 ^a^	14.5 ± 13.5 ^a^	17.1 ± 16.8 ^a^	9.6 ± 6.7 ^a^	11.8 ± 7.1 ^a^	8.8 ± 7.2 ^a^	8.5 ± 7.3 ^a^	0.6
FRO	3.1 ± 3.6 ^d^	11.0 ± 4.2 ^cd^	27.0 ± 10.9 ^a^	21.5 ± 6.8 ^ab^	18.8 ± 7.2 ^abc^	15.1 ± 3.8 ^bc^	12.1 ± 4.9 ^bcd^	11.8
CO	1.3 ± 2.3 ^e^	38.0 ± 8.3 ^bc^	27.0 ± 13.8 ^c^	48.4 ± 15.7 ^ab^	24.3 ± 6.0 ^cd^	56.8 ± 16.8 ^a^	9.5 ± 3.6 ^de^	26.7
BT	3.4 ± 7.6 ^d^	47.0 ± 8.6 ^b^	22.6 ± 11.3 ^c^	59.1 ± 13.8 ^b^	61.9 ± 11.7 ^b^	81.6 ± 10.1 ^a^	62.5 ± 11.8 ^b^	48.3
BT2	0.3 ± 0.5 ^b^	62.9 ± 17.4 ^a^	23.4 ± 10.6 ^b^	56.6 ± 14.2 ^a^	65.9 ± 14.5 ^a^	73.8 ± 17.3 ^a^	59.8 ± 22.1 ^a^	24.8
TF	23.5 ± 5.2 ^cd^	37.1 ± 7.7 ^bcd^	22.3 ± 4.2 ^d^	50.3 ± 12.1 ^ab^	63.6 ± 10.9 ^a^	59.8 ± 18.1 ^a^	41.3 ± 15.6 ^bc^	15.9
CF	0.1 ± 0.4 ^c^	47.4 ± 17.1 ^ab^	37.8 ± 20.1 ^b^	56.6 ± 12.8 ^ab^	57.1 ± 16.4 ^ab^	69.3 ± 21.5 ^a^	39.5 ± 17.6 ^b^	14.6
Hardness	29.8 ± 21.2 ^d^	64.5 ± 16.2 ^abc^	47.1 ± 18.5 ^cd^	75.1 ± 10.7 ^ab^	61.3 ± 23.5 ^abc^	85.6 ± 14.3 ^a^	56.3 ± 18.6 ^bcd^	8.2
Roughness	28.1 ± 16.5 ^c^	61.4 ± 20.9 ^ab^	43.1 ± 19.1 ^bc^	70.1 ± 20.0 ^ab^	58.4 ± 18.7 ^ab^	80.9 ± 12.4 ^a^	50.9 ± 14.6 ^bc^	7.8
Crunchiness	76.1 ± 19.3 ^a^	45.1 ± 8.3 ^c^	65.6 ± 7.4 ^ab^	45.8 ± 16.1 ^c^	50.9 ± 13.7 ^bc^	48.3 ± 5.9 ^bc^	57.9 ± 5.5 ^abc^	7.5
Saliva absorption	76.0 ± 16.0 ^a^	42.5 ± 10.8 ^b^	35.9 ± 5.7 ^bc^	29.9 ± 4.7 ^bc^	30.9 ± 6.4 ^bc^	25.6 ± 8.4 ^c^	66.0 ± 5.9 ^a^	37.2
Adhesiveness	47.5 ± 6.9 ^b^	57.3 ± 18.5 ^ab^	56.5 ± 9.2 ^ab^	62.5 ± 16.4 ^ab^	49.9 ± 14.5 ^b^	72.8 ± 19.9 ^a^	47.3 ± 10.9 ^b^	3.2

## Data Availability

Authors can confirm that all relevant data are included in the article and/or its Supplementary Material files. The datasets generated during and/or analyzed during the current study are available from the corresponding author on reasonable request.

## Supplementary Materials

The following supporting information can be downloaded at: https://www.mdpi.com/article/10.3390/foods11162393/s1, Table S1: List of sensory properties, descriptors with description and final markings on the scale used for sensory evaluation of snack products; Table S2: Consumption of specific mechanical energy (SME) during extrudates production; Table S3: Values of initial temperature (To), peak temperature (Tp), final temperature (Tv) and enthalpy (ΔH) of initial non-extruded blends (with CRF content of 20; 24.1; 30; 34.9 and 40%), and raw materials (chicory root and rice flour); Table S4: Values of initial temperature (To), peak temperature (Tp), final gelatinization temperature (Tv) and enthalpy (ΔH) of extrudates; Table S5: ANN summary (performance and errors), for training, testing and validation cycles; Table S6: The “goodness of fit” tests for the developed ANN model.

## References

[1] do Carmo C.S., Varela P., Poudroux C., Dessev T., Myhrer K., Rieder A., Zobel H., Sahlstrøm S., Knutsen S.H. (2019). The impact of extrusion parameters on physicochemical, nutritional and sensorial properties of expanded snacks from pea and oat fractions. LWT Food Sci. Technol..

[2] Brennan M.A., Derbyshire E., Tiwari B.K., Brennan C.S. (2013). Ready-to-eat snack products: The role of extrusion technology in developing consumer acceptable and nutritious snacks. Int. J. Food Sci. Technol..

[3] Grasso S. (2020). Extruded snacks from industrial by-products: A review. Trends Food Sci. Technol..

[4] Xu E., Pan X., Wu Z., Long J., Li J., Xu X., Jin Z., Jiao A. (2016). Response surface methodology for evaluation and optimization of process parameter and antioxidant capacity of rice flour modified by enzymatic extrusion. Food Chem..

[5] KC Y., Rajbanshi R., Katuwal N., Dhungana P., Subba D. (2021). Process optimization for yam flour incorporated in expanded extrudates. Int. J. Food Prop..

[6] Kolniak-Ostek J., Kita A., Pęksa A., Wawrzyniak A., Hamułka J., Jeznach M., Danilčenko H., Jariene E. (2017). Analysis of the content of bioactive compounds in selected flours and enriched extruded corn products. J. Food Compos. Anal..

[7] Perović J., Tumbas Šaponjac V., Kojić J., Krulj J., Moreno D.A., García-Viguera C., Bodroža-Solarov M., Ilić N. (2021). Chicory (*Cichorium intybus* L.) as a food ingredient—Nutritional composition, bioactivity, safety, and health claims: A review. Food Chem..

[8] Pouille C.L., Jegou D., Dugardin C., Cudennec B., Ravallec R., Hance P., Rambaud C., Hilbert J.L., Lucau-Danila A. (2020). Chicory root flour—A functional food with potential multiple health benefits evaluated in a mice model. J. Funct. Foods.

[9] Saeed M., El-Hack M.E.A., Alagawany M., Arain M.A., Arif M., Mirza M.A., Naveed M., Chao S., Sarwar M., Sayaba M. (2017). Chicory (*Cichorium intybus*) herb: Chemical composition, pharmacology, nutritional and healthical applications. Int. J. Pharmacol..

[10] Kaur N., Gupta A.K. (2002). Applications of inulin and oligofructose in health and nutrition. J. Biosci..

[11] Massoud I., Amin A., Elgindy A.A. (2009). Chemical and technological studies on chicory (*Cichorium intybus* L.) and its applications in some functional food. Int. J. Adv. Agric. Res..

[12] Jeong D., Kim D.-H., Oh Y.-T., Chon J.-W., Kim H., Jeong D.-K., Kim H., Kim Y.-G., Song K.-Y., Kim Y.-J. (2017). Production of bioactive yoghurt containing *Cichorium intybus* L. (chicory) extract-preliminary study. J. Milk Sci. Biotechnol..

[13] Kumar D., DC R., Alam T., Sawant P. (2018). Effect of dried chicory root extract on sensory and physical characteristics of yoghurt-ice cream with addition of buttermilk using response surface methodology. J. Food Dairy Technol..

[14] Kojić J.S., Ilić N.M., Kojić P.S., Pezo L.L., Banjac V.V., Krulj J.A., Bodroža Solarov M.I. (2019). Multiobjective process optimization for betaine enriched spelt flour based extrudates. J. Food Process Eng..

[15] Kowalski R.J., Pietrysiak E., Ganjyal G.M. (2021). Optimizing screw profiles for twin-screw food extrusion processing through genetic algorithms and neural networks. J. Food Eng..

[16] Pasqualone A., Costantini M., Labarbuta R., Summo C. (2021). Production of extruded-cooked lentil flours at industrial level: Effect of processing conditions on starch gelatinization, dough rheological properties and techno-functional parameters. LWT.

[17] Vujčić I., Mašić S. (2021). Preservation of hemp flour using high-energy ionizing radiation: The effect of gamma radiation on aflatoxin inactivation, microbiological properties, and nutritional values. J. Food Process Preserv..

[18] Perović J., Kojić J., Krulj J., Pezo L., Tumbas Šaponjac V., Ilić N., Bodroža-Solarov M. (2022). Inulin determination by an improved HPLC-ELSD method. Food Anal. Methods.

[19] Vallée M., Lu X., Narciso J.O., Li W., Qin Y., Brennan M.A., Brennan C.S. (2017). Physical, predictive glycaemic response and antioxidative properties of black ear mashroom (*Auricularia auricula*) extrudates. Plant Foods Hum. Nutr..

[20] Han Y.J., Tra Tran T.T., Man Le V.V. (2018). Corn snack with high fiber content: Effects of different fiber types on the product quality. LWT Food Sci. Technol..

[21] Operation Instructions Manual from Mettler Toledo Density Kit. https://www.mt.com/hk/en/home/library/operating-instructions/laboratory-weighing/NC_density_OI.html.

[22] Ačkar Đ., Jozinović A., Babić J., Miličević B., Panak Balentić J., Šubarić D. (2018). Resolving the problem of poor expansion in corn extrudates enriched with food industry by-products. Innov. Food Sci. Emerg. Technol..

[23] Smarzyński K., Sarbak P., Kowalczewski P.Ł., Różańska M.B., Rybicka I., Polanowska K., Fedko M., Kmiecik D., Masewicz Ł., Nowicki M. (2021). Low-Field NMR study of shortcake biscuits with cricket powder, and their nutritional and physical characteristics. Molecules.

[24] Wang P., Yang Q., Zheng D., Wang Q., Wang N., Saleh A.S.M., Zhu M., Xiao Z. (2018). Physicochemical and antioxidant properties of rice flour based extrudates enriched with stabilized rice bran. Starch-Stärke.

[25] Höglund E., Eliasson L., Oliveira G., Almli V.L., Sozer N., Alminger M. (2018). Effect of drying and extrusion processing on physical and nutritional characteristics of bilberry press cake extrudates. LWT Food Sci. Technol..

[26] Heidenreich S., Jaros D., Rohm H., Ziems A. (2004). Relationship between water activity and crispness of extruded rice crisps. J. Textural Stud..

[27] Arivalagan M., Manikantan M.R., Yasmeen A.M., Sreejith S., Balasubramanian D., Hebbar K.B., Kanade S.R. (2018). Physiochemical and nutritional characterization of coconut (*Cocos nucifera* L.) haustorium based extrudates. LWT Food Sci. Technol..

[28] Voca N., Pezo L., Peter A., Suput D., Loncar B., Kricka T. (2021). Modelling of corn kernel pre-treatment, drying and processing for ethanol production using artificial neural networks. Ind. Crops Pro..

[29] Bisharat G.I., Oikonomopoulou V.P., Panagiotou N.M., Krokida M.K., Maroulis Z.B. (2013). Effect of extrusion conditions on the structural properties of corn extrudates enriched with dehydrated vegetables. Food Res. Int..

[30] Seth D., Badwaik L.S., Ganapathy V. (2015). Effect of feed composition, moisture content and extrusion temperature on extrudate characteristics of yam-corn-rice based snack food. J. Food Sci. Technol..

[31] Zhao Y., Zhao C., Tang X., Zhou J., Li H., Zhang H., Liu J. (2021). Physicochemical properties and microstructure of corn flour–cellulose fiber extrudates. Food Sci. Nutr..

[32] Tsokolar-Tsikopoulos K.C., Katsavou I.D., Krokida M.K. (2015). The effect of inulin addition on structural and textural properties of extruded products under several extrusion conditions. J. Food Sci. Technol..

[33] Nascimento T.A., Calado V., Carvalho C.W.P. (2017). Effect of Brewer’s spent grain and temperature on physical properties of expanded extrudates from rice. LWT Food Sci. Technol..

[34] Moraru C.I., Kokini J.L. (2003). Nucleation and expansion during extrusion and microwave heating of cereal foods. Compr. Revi. Food Sci. Food Saf..

[35] Rytel E., Pęksa A., Tajner-Czopek A., Kita A., Zięba T., Gryszkin A. (2013). Effect of addition of protein preparations on the quality of extruded maize extrudates. J. Microbiol. Biotechnol. Food Sci..

[36] Kojić J., Krulj J., Peić Tukuljac L., Jevtić Mučibabić R., Cvetković B., Kojić P., Ilić N. The effect of extrusion conditions on the bulk density of spelt wholegrain snack product. Proceedings of the VII Internation al Congress “Engineering, Environment and Materials in Process Industry” EEM2021.

[37] Alam M.S., Pathania S., Sharma A. (2016). Optimization of the extrusion process for development of high fibre soybean-rice ready-to-eat snacks using carrot pomace and cauliflower trimmings. LWT Food Sci. Technol..

[38] Jiamjariyatam R. (2017). Microwavable expanded-snack from native rice starch: Influence of inulin and amylose content. Int. Food Res. J..

[39] El-Samahy S.K., Abd El-Hady E.A., Habiba R.A., Moussa-Ayoub T.E. (2007). Some functional, chemical, and sensory characteristics of cactus pear rice-based extrudates. J. Prof. Assoc. Cactus Dev..

[40] Makowska A., Baranowska H.M., Michniewicz J., Chudy S., Kowalczewski P.Ł. (2017). Triticale extrudates—Changes of macrostructure, mechanical properties and molecular water dynamics during hydration. J. Cereal Sci..

[41] Yağcı S., Altan A., Doğan F. (2020). Effects of extrusion processing and gum content on physicochemical, microstructural and nutritional properties of fermented chickpea-based extrudates. LWT Food Sci. Technol..

[42] Hegazy H.S., El-Fath A., El-Bedawey A., Rahma E.-S.H., Gaafar A.M. (2017). Effect of extrusion rocesss on nutritional, functional properties and antioxidant activity of germinated chickpea incorporated corn extrudates. Am. J. Food Sci. Nutr. Res..

[43] Morris C., Morris G.A. (2012). The effect of inulin and fructo-oligosaccharide supplementation on the textural, rheological and sensory properties of bread and their role in weight management: A review. Food Chem..

[44] Rayan A.M., Morsy N.E., Youssef K.M. (2018). Enrichment of rice-based extrudates with Cactus Opuntia dillenii seed powder: A novel source of fiber and antioxidants. J. Food Sci. Technol..

[45] Dalbhagat C.G., Mahato D.K., Mishra H.N. (2019). Effect of extrusion processing on physicochemical, functional and nutritional characteristics of rice and rice-based products: A review. Trends Food Sci. Technol..

[46] Bakalov I.Y., Petrova T.V., Ruskova M.M., Karadzhova K.D.K., Penov N.D. (2016). The effect of extrusion variables on the colour of bean-based extrudates. Bulg. Chem. Commun..

[47] Kaur A., Kaur S., Singh M., Singh N., Shevkani K., Singh B. (2014). Effect of banana flour, screw speed and temperature on extrusion behaviour of corn extrudates. J. Food Sci. Technol..

[48] Bertoft E. (2017). Understanding Starch Structure: Recent Progress. Agronomy.

[49] Kiumarsi M., Shahbazi M., Yeganehzad S., Majchrzak D., Lieleg O., Winkeljann B. (2019). Relation between structural, mechanical and sensory properties of gluten-free bread as affected by modified dietary fibers. Food Chem..

[50] Samyor D., Deka S.C., Das A.B. (2018). Effect of extrusion conditions on the physicochemical and phytochemical properties of red rice and passion fruit powder based extrudates. J. Food Sci. Technol..

[51] da Silva E.M.M., Ascheri J.L.R., de Carvalho C.W.P., Takeiti C.Y., de Berrios J.J. (2014). Physical characteristics of extrudates from corn flour and dehulled carioca bean flour blend. LWT Food Sci. Technol..

[52] Poliszko N., Kowalczewski P.Ł., Rybicka I., Kubiak P., Poliszko S. (2019). The effect of pumpkin flour on quality and acoustic properties of extruded corn snacks. J. Verbrauch. Lebensm..

