# Supplementary material for: Development of a Novel Rice-Based Snack Enriched with Chicory Root: Physicochemical and Sensory Properties
Source: Foods. 2022 Aug 9;11(16):2393. doi: 10.3390/foods11162393 (PMC9407501; doi:10.3390/foods11162393)
Supplement: Supplementary file 1 [file foods-11-02393-s001.zip › foods-1826947-supplementary.pdf]

Supplementary material

Table S1. List of sensory properties, descriptors with description and final markings on the scale used for sensory evaluation of snack products.

Sensory properties	Descriptor	Definitions	End anchors on the scale
**Appereance**			
	Color shade	The color shade of the product from white to brown is evaluated according to the submitted scale.	White → Brown
	Color uniformity	Color uniformity The degree of uniform color distribution.	Homogeneous → Non-homogeneous
	Pore shape at cross section	Description of the shape of the cavities in the cross-section of the sample.	Round → Elliptical
	Appearance of cross-sectional pores	Description of the appearance of the cavities at the cross-section of the sample.	Puffy → Waxy
	Pore size at cross section	Relative size of cavities at the cross section of the sample.	Small → Large
**Odor**			
	Total odor intensity (TO)	The intensity of the total odor of the product assessed by direct smelling.	Weak → Intensive
	The intensity of the odor of rice (RO)	The intensity of the odor that comes from cooked rice.	Weak → Intensive
	Odor intensity on fried rice (FRO)	Intensity of the odor originating from rice that has been sufficiently thermally treated to caramelize part of the starch and sugar present.	Weak → Intensive
	The intensity of the odor on chicory (CO)	The intensity of the smell that comes from chicory poured over warm water.	Weak → Intensive
**Taste**			
	Bitterness (BT)	Intensity of bitter taste associated with caffeine solution.	Weak → Intensive
**Flavor**			
	Total flavor intensity (TF)	The total flavor intensity of the product assessed during/after manipulation of the sample in the mouth.	Weak → Intensive
	Flavor intensity on chicory (CF)	The intensity of flavor associated with the feeling of chicory assessed during/after manipulation of the sample in the mouth.	Weak → Intensive
**Texture in the mouth**			
	Hardness	The force required for teeth to penetrate a sample.	Soft → Hard
	Rougness	The degree to which the mass is coarse and has large particles.	Gentle → Rough
	Crunchiness	The sound and force with which a sample breaks and cracks.	Gooey → Crispy

Table S1. Continuation of the Table S1.

Sensory properties	Descriptor	Definitions	End anchors on the scale
	Saliva absorption	The amount of saliva absorbed by a sample.	None → Completely
	Adhesiveness	The force required to remove the sample from the molar.	Not at all adhesive → Very adhesive
**Subsequent impressions**			
	Bitter taste after 2 min (BT2)	Intensity of bitter taste sensation two minutes after sample swallowing.	Weak → Intensive

Table S2. Consumption of specific mechanical energy (SME) during extrudates production.

S.no.	M, %	SS, rpm	P, %	SME (Wh/kg)
1	21.2	820	35.9	147.5
2	19.4	700	30.0	159.7
3	17.6	820	35.9	193.3
4	19.4	700	30.0	137.6
5	21.2	820	24.1	130.8
6	22.5	700	30.0	124.2
7	19.4	900	30.0	166.0
8	17.6	820	24.1	175.7
9	19.4	700	30.0	141.9
10	21.2	580	24.1	117.1
11	16.3	700	30.0	166.5
12	19.4	700	40.0	157.0
13	19.4	500	30.0	121.7
14	19.4	700	30.0	154.1
15	17.6	580	24.1	145.7
16	21.2	580	35.9	123.2
17	17.6	580	35.9	168.0
18	19.4	700	20.0	143.1
19	19.4	700	30.0	146.4
20	19.4	700	30.0	143.9

S.no. – Sample number; M – moisture content, SS – screw speed, P – chicory root flour (CRF) content, SME – specific mechanical energy.

Table S3. Values of initial temperature (To), peak temperature (Tp), final temperature (Tv) and enthalpy (ΔH) of initial non-extruded blends (with CRF content of 20; 24.1; 30; 34.9 and 40%), and raw materials (chicory root and rice flour).

n.e.b./r.m., %	Peak I				Peak II			
	To, ˚C	Tp, ˚C	Tv, ˚C	ΔH, J/g	To, ˚C	Tp, ˚C	Tv, ˚C	ΔH, J/g
20	45.96±0.15	49.80±0.10	55.26±0.15	4.50±0.03	68.40±0.20	73.40±0.40	78.30±0.40	3.12±0.20
24.1	43.60±0.40	47.36±0.35	53.46±0.35	4.21±0.02	68.00±0.40	73.20±0.20	79.10±0.30	2.57±0.07
30	44.06±0.25	47.43±0.35	54.86±0.35	4.21±0.01	70.4±0.40	74.80±0.50	80.10±0.40	2.48±0.19
35.9	47.53±0.15	51.13±0.15	53.86±0.63	3.80±0.12	66.20±0.40	71.20±0.20	82.60±1.30	1.45±0.24
40	44.27±0.06	47.16±0.21	54.47±0.46	2.57±0.22	67.70±0.50	70.80±0.30	77.30±0.30	0.68±0.09
Chicory root	/	/	/	/	/	/	/	/
Rice flour	44.93 ±0.50	48.46 ±0.35	55.63 ±0.40	5.13 ±0.59	66.1 ±0.50	71.1 ±0.10	76.7 ±0.80	3.17 ±0.04

n.e.b. – non-extruded blend; r.m. – raw material; To - initial temperature, Tp - peak temperature; Tv - final gelatinization temperature; and ΔH - enthalpy of extrudates.

Table S4. Values of initial temperature (To), peak temperature (Tp), final gelatinization temperature (Tv) and enthalpy (ΔH) of extrudates.

S.no.	M, %	SS, rpm	P, %	To, ˚C	Tp, ˚C	Tv, ˚C	ΔH, J/g
1	21.2	820	35.9	45.53±0.41	48.76±0.50	54.40±0.85	1.24±0.03
2	19.4	700	30	44.63±0.17	48.2±0.86	54.56±0.89	3.26±0.04
3	17.6	820	35.9	45.50±0.17	48.76±0.15	54.13±0.80	1.46±0.02
4	19.4	700	30	43.90±0.10	47.60±0.20	53.93±0.06	3.20±0.01
5	21.2	820	24.1	45.13±0.38	48.50±0.53	53.60±0.56	1.81±0.05
6	22.5	700	30	44.73±0.35	47.66±0.21	53.23±0.96	1.64±0.04
7	19.4	900	30	44.03±0.15	47.30±0.36	53.10±0.30	2.33±0.02
8	17.6	820	24.1	44.50±0.36	47.70±0.26	52.50±0.56	1.27±0.10
9	19.4	700	30	44.66±0.06	48.16±0.11	55.60±0.36	3.24±0.03
10	21.2	580	24.1	44.70±0.26	48.00±0.26	54.26±0.65	3.17±0.05
11	16.3	700	30	44.26±0.25	47.60±0.26	54.33±0.77	2.47±0.01
12	19.4	700	40	44.43±0.40	47.70±0.30	54.23±0.46	3.57±0.02
13	19.4	500	30	44.13±0.12	47.36±0.15	53.40±0.35	2.39±0.05
14	19.4	700	30	46.13±0.40	49.23±0.32	56.36±0.85	3.24±0.01
15	17.6	580	24.1	45.03±0.30	48.30±0.20	53.73±0.15	1.91±0.04
16	21.2	580	35.9	44.33±0.35	47.53±0.15	53.63±0.31	2.20±0.04
17	17.6	580	35.9	45.13±0.15	48.06±0.15	53.70±0.26	1.35±0.06
18	19.4	700	20	45.03±0.06	48.20±0.20	53.53±0.59	1.64±0.03
19	19.4	700	30	44.43±0.51	48.00±0.50	54.56±0.90	3.25±0.05
20	19.4	700	30	44.50±0.26	47.80±0.26	54.63±0.38	3.26±0.03
CS	18.0	800	0	61.31±0.33	62.81±0.46	84.43±0.51	1.28±0.05

S.no – sample number; M – moisture content; SS – screw speed; P – chicory root flour (CRF) content; To - initial temperature; Tp - peak temperature; Tv - final gelatinization temperature; ΔH - enthalpy of extrudates; CS – control sample.

Table S5. ANN summary (performance and errors), for training, testing and validation cycles.

Network name	Performance			Error			Training algorithm	Error function	Hidden activation	Output activation
	Train.	Test.	Valid.	Train.	Test.	Valid.				
MLP 3-10-11	0.999	0.997	0.998	1384.4	1583.3	1511.7	BFGS 10000	SOS	Logistic	Identity

*Performance term represent the coefficients of determination, while error terms indicate lack of data for the ANN model. Train. – training; Test. – testing; Valid. – validation cycles.

Table S6. The "goodness of fit" tests for the developed ANN model.

	χ^2	RMSE	MBE	MPE	SSE	AARD	R^2
EI	0.001	0.017	0.000	0.561	0.006	0.644	0.999
BD	19.0	2.850	-0.006	0.969	170.6	100.4	0.998
D	349.5	12.239	-0.014	1.914	3145.7	417.1	0.992
V	6068.5	50.998	0.036	3.186	54616.4	875.7	0.990
L*	1.194	0.715	0.000	0.719	10.748	12.716	0.991
a*	0.060	0.160	0.000	2.328	0.539	3.580	0.985
b*	0.059	0.159	0.000	0.524	0.534	2.790	0.998
ΔE	0.846	0.602	0.000	1.956	7.612	6.795	0.970
DG	0.132	0.238	-0.001	0.563	1.190	4.543	1.000
Ci	0.030	0.113	0.000	7.481	0.270	4.906	0.992
Hardness	21.494	3.035	-0.005	2.000	193.447	43.347	0.994

χ^2- reduced chi-square, RMSE - root mean square error, MBE - mean bias error, MPE - mean percentage error, SSE - sum of squared errors, AARD - average absolute relative deviation, R^2 - coefficient of determination, EI – expansion index, BD – bulk density, D – density, V – volume, DG – degree of gelatinization, Ci – crispiness index, CS – control sample.
